# Supplementary material for: Luminal epithelial cells integrate variable responses to aging into stereotypical changes that underlie breast cancer susceptibility
Source: eLife. 2024 Nov 15;13:e95720. doi: 10.7554/eLife.95720 (PMC11723586; doi:10.7554/eLife.95720)
Supplement: MDAR checklist [file elife-95720-mdarchecklist1.pdf]

eLife

Westbrook Centre
Milton Road
Cambridge CB4 1YG, UK

P 01223 855340
W elifesciences.org
T @elife

Materials Design Analysis Reporting (MDAR) Checklist for Authors

The MDAR framework establishes a minimum set of requirements in transparent reporting mainly applicable to studies in the life sciences.

eLife asks authors to **provide detailed information within their article** to facilitate the interpretation and replication of their work. Authors can also upload supporting materials to comply with relevant reporting guidelines for health-related research (see EQUATOR Network), life science research (see the BioSharing Information Resource), or animal research (see the ARRIVE Guidelines and the STRANGE Framework; for details, see *eLife*'s Journal Policies). Where applicable, authors should refer to any relevant reporting standards materials in this form.

For all that apply, please note **where in the article** the information is provided. Please note that we also collect information about data availability and ethics in the submission form.

Materials:

Newly created materials	Indicate where provided: section/figure legend	N/A
The manuscript includes a dedicated "materials availability statement" providing transparent disclosure about availability of newly created materials including details on how materials can be accessed and describing any restrictions on access.	See Declarations, Availability of data and materials section, pg. 51.<br>See Supplementary Methods, Materials Availability section, pg. 2.	

Antibodies	Indicate where provided: section/figure legend	N/A
For commercial reagents, provide supplier name, catalogue number and RRID, if available.	See Methods, Method Details section, pg. 40.<br>For full details, see Supplementary Methods, Method Details section, pg. 6.	

DNA and RNA sequences	Indicate where provided: section/figure legend	N/A
Short novel DNA or RNA including primers, probes: Sequences should be included or deposited in a public repository.	See Methods, Method Details section, pg. 40.<br>For full details, see Supplementary Methods, Materials Availability section, pg. 2.	

Cell materials	Indicate where provided: section/figure legend	N/A

Cell lines: Provide species information, strain. Provide accession number in repository OR supplier name, catalog number, clone number, OR RRID.		N/A
Primary cultures: Provide species, strain, sex of origin, genetic modification status.	See Figure 1 – table supplement 1A<br>See Methods, Experimental Model and Subject Details section, pg. 38<br>For full details, see Supplementary Methods, Experimental Model and Subject Details section, pg. 3.	

**Experimental animals**	**Indicate where provided: section/figure legend**	**N/A**
Laboratory animals or Model organisms: Provide species, strain, sex, age, genetic modification status. Provide accession number in repository OR supplier name, catalog number, clone number, OR RRID.		N/A
Animal observed in or captured from the field: Provide species, sex, and age where possible.		N/A

**Plants and microbes**	**Indicate where provided: section/figure legend**	**N/A**
Plants: provide species and strain, ecotype and cultivar where relevant, unique accession number if available, and source (including location for collected wild specimens).		N/A
Microbes: provide species and strain, unique accession number if available, and source.		N/A

**Human research participants**	**Indicate where provided: section/figure legend) or state if these demographics were not collected**	**N/A**
If collected and within the bounds of privacy constraints report on age, sex, gender and ethnicity for all study participants.		N/A

Design:

**Study protocol**	**Indicate where provided: section/figure legend**	**N/A**

If the study protocol has been pre-registered, provide DOI. For clinical trials, provide the trial registration number OR cite DOI.		N/A

**Laboratory protocol**	**Indicate where provided: section/figure legend**	**N/A**
Provide DOI OR other citation details if detailed step-by-step protocols are available.	See Methods, Experimental Model and Subject Details section, pg. 38<br>For full details, see Supplementary Methods, Experimental Model and Subject Details section, pg. 3.	

**Experimental study design (statistics details) ***		
**For in vivo studies: State whether and how the following have been done**	**Indicate where provided: section/figure legend. If it could have been done, but was not, write "not done"**	**N/A**
Sample size determination	See Methods, Materials Design Analysis section: Sample size estimation, pg. 39<br>For full details, see Supplementary Methods, Materials Design Analysis section: Sample size estimation, pg. 5.	
Randomisation		N/A
Blinding		N/A
Inclusion/exclusion criteria	See Methods, Experimental Model and Subject Details section, pg. 38 and Method Details section, pg. 40.<br>For full details, see Supplementary Methods, Experimental Model and Subject Details section, pg. 3 and Method Details section, pg. 6.	

**Sample definition and in-laboratory replication**	**Indicate where provided: section/figure legend**	**N/A**
State number of times the experiment was replicated in the laboratory.	See Figure and Figure Legends for subject (n), sample (m) biological replicate, and technical replicate (l) size for each analysis or experiment.	

Define whether data describe technical or biological replicates.	See Methods, Materials Design Analysis section: Replicates, pg. 39.<br>For full details, see Supplementary Methods, Materials Design Analysis section: Replicates, pg. 4.	

**Ethics**	**Indicate where provided: section/submission form**	**N/A**
Studies involving human participants: State details of authority granting ethics approval (IRB or equivalent committee(s), provide reference number for approval.		N/A
Studies involving experimental animals: State details of authority granting ethics approval (IRB or equivalent committee(s), provide reference number for approval.		N/A
Studies involving specimen and field samples: State if relevant permits obtained, provide details of authority approving study; if none were required, explain why.		N/A

**Dual Use Research of Concern (DURC)**	**Indicate where provided: section/submission form**	**N/A**
If study is subject to dual use research of concern regulations, state the authority granting approval and reference number for the regulatory approval.		N/A

Analysis:

**Attrition**	**Indicate where provided: section/figure legend**	**N/A**
Describe whether exclusion criteria were pre-established. Report if sample or data points were omitted from analysis. If yes, report if this was due to attrition or intentional exclusion and provide justification.	See Methods, Method Details section, pg. 40.<br>For full details, see Supplementary Methods, Method Details section, pg. 6.	

**Statistics**	**Indicate where provided: section/figure legend**	**N/A**
Describe statistical tests used and justify choice of tests.	See Methods, Quantification and Statistical Analysis section, pg. 44<br>For full details, see Supplementary Methods, Quantification and	

	Statistical Analysis section. pg. 15.	

Data availability	Indicate where provided: section/submission form	N/A
For newly created and reused datasets, the manuscript includes a data availability statement that provides details for access (or notes restrictions on access).	See Declarations, Availability of data and materials section, pg. 51. See Supplementary Methods, Data and Code Availability section, pg. 2.	
When newly created datasets are publicly available, provide accession number in repository OR DOI and licensing details where available.	NCBI Gene Expression Omnibus, GSE182338 https://www.ncbi.nlm.nih.gov/geo/query/acc.cgi?acc=GSE182338	
If reused data is publicly available provide accession number in repository OR DOI, OR URL, OR citation.	(1) NCBI Gene Expression Omnibus, GSE102088; https://www.ncbi.nlm.nih.gov/geo/query/acc.cgi?acc=GSE102088 (downloaded via GEOquery package);<br>(2) NCBI Gene Expression Omnibus, GSE81540; https://www.ncbi.nlm.nih.gov/geo/query/acc.cgi?acc=GSE81540 (downloaded via GEOquery package);<br>(3) GDC Portal, TCGA BRCA Harmonized RNA-Seq FPKM data; https://portal.gdc.cancer.gov/ (downloaded via TCGAbiolinks package);<br>(4) GTEx Portal, GTEx Breast RNA-Seq count data; https://gtexportal.org/home/ (downloaded via recount3 pacakge)<br>(5) NCBI Gene Expression Omnibus, GSE161529; https://www.ncbi.nlm.nih.gov/geo/query/acc.cgi?acc=GSE161529 (provided as a Seurat object by the group of Dr. Andrea Bild, City of Hope)<br>(6) NCBI Gene Expression Omnibus, GSE174588; https://www.ncbi.nlm.nih.gov/geo/query/acc.cgi?acc=GSE174588 (provided as a Seurat object by Dr.Kai Kessenbrock, City of Hope)<br>(7) NCBI Gene Expression	

	Omnibus, GSE198732; https://www.ncbi.nlm.nih.gov/geo/query/acc.cgi?acc=GSE198732 (downloaded from NCBI GEO as a Seurat object)	

**Code availability**	**Indicate where provided: section/figure legend**	**N/A**
For any computer code/software/mathematical algorithms essential for replicating the main findings of the study, whether newly generated or re-used, the manuscript includes a data availability statement that provides details for access or notes restrictions.	See Declarations, Availability of data and materials section, pg. 51. See Supplementary Methods, Data and Code Availability section, pg. 2.	
Where newly generated code is publicly available, provide accession number in repository, OR DOI OR URL and licensing details where available. State any restrictions on code availability or accessibility.		N/A
If reused code is publicly available provide accession number in repository OR DOI OR URL, OR citation.	All R/Bioconductor packages and citation information are listed. See Methods pg. 38. Package versions are additionally listed in Supplementary Methods.	

Reporting:

The MDAR framework recommends adoption of discipline-specific guidelines, established and endorsed through community initiatives.

**Adherence to community standards**	**Indicate where provided: section/figure legend**	**N/A**
State if relevant guidelines (e.g., ICMJE, MIBBI, ARRIVE, STRANGE) have been followed, and whether a checklist (e.g., CONSORT, PRISMA, ARRIVE) is provided with the manuscript.		N/A

---

* We provide the following guidance regarding transparent reporting and statistics; we also refer authors to Ten common statistical mistakes to watch out for when writing or reviewing a manuscript.

Sample-size estimation

See Methods, Materials Design Analysis section: Sample-size estimation, pg. 39.
For full details, see Supplementary Methods, Materials Design Analysis section: Sample-size estimation, pg. 5.

Replicates

See Methods, Materials Design Analysis section: Replicates, pg. 39.
For full details, see Supplementary Methods, Materials Design Analysis section: Replicates, pg. 4.

Statistical reporting

See Methods, Materials Design Analysis section: Statistical reporting, pg. 40 and Quantification and Statistical Analysis section, pg. 44.
For full details, see Supplementary Methods, Materials Design Analysis section: Statistical reporting, pg. 6 and Quantification and Statistical Analysis section. pg. 15.

Group allocation

See Methods, Materials Design Analysis section: Group allocation, pg. 38.
For full details, see Supplementary Methods, Materials Design Analysis section: Group allocation, pg. 4.
